# Post-partum Thrombotic Thrombocytopenic Purpura with Puerperal Sepsis- A Case Report

**DOI:** 10.1016/j.amsu.2022.104828

**Published:** 2022-11-05

**Authors:** Bushra Majid, Syed Hassan Ahmed, Hanifa Majid

**Affiliations:** Dow University of Health Sciences, Karachi, Pakistan

**Keywords:** Thrombotic thrombocytopenic purpura, Thrombotic microangiopathy, Maternal health, Puerperal sepsis, Case report

## Abstract

**Introduction:**

Post-partum TTP is an uncommon thrombotic microangiopathy affecting about 1 in 200,000 pregnancies in contrast to preeclampsia and HUS, which have been reported commonly.

**Case presentation:**

We report a case of a postpartum TTP following purpureal sepsis. The patient was brought with per vaginal bleed, vomiting, chest pain, yellow discoloration of sclera, and abdominal discomfort following a spontaneous vaginal delivery two days back at a hospital.

**Clinical findings and investigations:**

The workup revealed anemia and thrombocytopenia with deranged PT/INR. The renal profile deteriorated over one day and she also developed psychosis. Additionally, schistocytes were observed on the peripheral blood smear.

**Interventions and outcome:**

The patient was subsequently treated with dialysis followed by plasmapheresis in addition to the antibiotics after the diagnosis of TTP and made a complete recovery.

**Relevance and impact:**

25% of TTP occurs in the intra or postpartum period. It is thus pivotal to keep it among differentials and intervened timely to reduce morbidity and mortality. Vigilance is required to prevent any relapse in subsequent pregnancies.

## Highlights

• Thrombotic thrombocytopenic purpura arising in postpartum is not an oft-sighted entity following puerperal sepsis.
• Pregnant women are more susceptible to developing Thrombotic Thrombocytopenic purpura and Immune Thrombocytopenic purpura.
• Puerperal Sepsis may complicate into systemic manifestations if not treated promptly.
• TTP is considered a medical emergency in pregnancy and postpartum.
• Dialysis and plasmapheresis remain the mainstay of treatment.

## 1 Introduction

Thrombotic microangiopathy disorders are characterized by microangiopathic hemolytic anemia (MAHA), severe thrombocytopenia, and organ injury due to clot formation in the microvasculature. In pregnancy, microangiopathy disorders are considered a medical emergency, and they most commonly manifest as preeclampsia or HELLP syndrome (hemolysis, elevated liver enzyme levels, and low platelet levels) [1]. Thrombotic thrombocytopenic purpura (TTP) is a rare thrombotic microangiopathy in pregnancy, with an approximate occurrence of 1 in 200,000, and is considered to be a medical emergency that needs prompt diagnosis and treatment [2]. Around 25% of TTP occurs in the intra or postpartum period [1]. This disorder is characterized by the deposition of fibrin in the microvasculature of the organs leading to endothelial swelling and ultimate organ dysfunction and even failure. Kidney function is seen to be normal or just mildly affected [3]. If prompt timely treatment and diagnosis are not made, it can lead to a 95% mortality rate in pregnant women and 80% mortality in fetuses [4]. Hence it becomes very important to address this life-threatening phenomenon. In this case report, we present a case of a 27-year-old female who developed severe AKI secondary to TTP after she had delivered a healthy baby and went on to have dialysis and plasmapheresis before making a complete recovery. This case has been reported in accordance with the SCARE 2020 (Surgical Case Report) guidelines [5].

## 2 Presentation of case

We present the case of a 27-year-old female, housewife, parity 2^+1^ with no known co-morbid and having a full-term spontaneous vaginal delivery at another hospital two days back, attended the ER with complaints of per vaginal bleed, vomiting, chest pain, yellow discoloration of sclera, and drowsiness for one day. She developed bleeding from the vagina 4 h after delivery, acute in onset, progressive, and associated with abdominal discomfort. This was followed by a few episodes of vomiting, that were non-projectile, postprandial, and not bile or blood-stained. The patient also complained of chest pain that was acute in onset, dull in character with no radiation but associated with palpitations and shortness of breath. Furthermore, the patient developed yellow discoloration of sclera and drowsiness but there was no accompanying fever or rash. The patient's family history was not significant for a similar condition or any hereditary disorder. Also, her past medical and surgical history was unremarkable and there was also no history of allergy. The patient denied taking any medication currently or previously. Her second pregnancy ended in spontaneous abortion at 20 weeks of gestation unlike her first and third where the babies were born full term via normal vaginal delivery.

On examination, she looked anxious and lethargic but was oriented to time, place, and person. She appeared anemic and jaundiced. Her receiving vitals were as follows: Blood pressure 105/75, pulse 140 beats per minute (bpm), temperature 98F, respiratory rate 25 breaths per minute, oxygen saturation of 97% on room air. All relevant laboratory investigations and Arterial Blood Gas (ABG) findings have been summarized in Table 1, Table 2.

**Table 1:** Results of laboratory Investigation.

Laboratory Investigations	Normal Values	On Admission	After 12 hours	Post-transfusion	Post-dialysis
Hb (Hemoglobin)	Males: 13.2–16.6 g/dL Females: 11.6–15 g/dL	7.2	5.9	9.1	–
TLC (Total leukocyte count)	4000 – 11,000 cells/mm3	30.8	37.2	50.8	–
Platelets	150,000–450,000 platelets/uL	19	19	47	–
PT/APTT (Prothrombin time/Activated partial thromboplastin time)	60 – 70 s/30 – 40 s	36.4/88.5	28.7/77.9	–	–
INR (International normalized ratio)	≤ 1.1	2.7	2.2	–	–
BUN (Blood urea nitrogen)	6–20 mg/dL	27	–	67	80
Creatinine	Males: 0.7–1.3 mg/dL (61.9–114.9 μmol/L) Females: 0.6–1.1 mg/dL (53–97.2 μmol/L)	–	–	4.77	3.82
Na (Sodium)	135-145 mEq/L	138	–	142	139
Cl (Chloride)	96-106 mEq/L	108	–	108	105
K (Potassium)	3.6–5.2mmol/L	4.5	–	3.5	3.5
Mg (Magnesium)	1.3-2.1 mEq/L (0.65–1.05 mmol/L)	–	–	2.9	–
Ca (Calcium)	8.5–10.5 mg/dL	–	–	7.0 (corrected Ca = 8.5)	–
PO4 (Phosphorus)	2.8–4.5 mg/dL	–	–	7.7	5.6
HCO3 (Bicarbonate)	22-29 mEq/L	15	–	–	–
Total Bilirubin	0.1–1.2 mg/dL	7.22	–	6.01	–
Direct Bilirubin	<0.3 mg/dL	4.87	–	4.41	–
SGPT (Serum glutamic pyruvic transaminase)	7–56 units/ L of blood serum	6	–	231	–
SGOT (Serum glutamic-oxaloacetic transaminase)	8–45 units/L of serum	524	–	602	–
ALK. PHOS (Alkaline phosphatase)	44–147 IU/L	109	–	115	–
γ-GT (Gamma-glutamyl transpeptidase)	5–40 U/L	30	–	31	–
LDH (Lactate dehydrogenase)	105–333 IU/L	4755	–		–
CRP (C-Reactive protein)	<10 mg/L	189.2	–		–
PCT (Procalcitonin)	<0.1 ng/mL	>100	–		–
FDP (mg/ml) (Fibrin degradation products)	<10 mcg/mL (10 mg/L)	>20	–		–

Hb: Hemoglobin; TLC: Total Leukocyte Count; INR: International Normalized Ratio; BUN: Blood Urea Nitrogen; SGPT: Serum Glutamic Pyruvic Transaminase; SGOT: Serum Glutamic Oxaloacetic Transaminase; Gamma-GT: Gamma-Glutamyl Transferase; LDH: Lactate Dehydrogenase; CRP: C-Reactive Protein; PCT: Pro-Calcitonin; FDP: Fibrin Degradation Products.

**Table 2:** Results of Arterial Blood Gases (ABGs).

Parameters	Normal values	Value
pH (power of hydrogen)	7.35–7.45	7.42
pCO2 (partial pressure of carbon dioxide)	35–45 mmHg	22.9
p02 (partial pressure of oxygen)	75–100 mmmmHg	129.6
HC03 (bicarbonate)	22 to 26 mEq/L	15
SpO2 (Saturation of oxygen)	≥95%	99.1

Viral markers including Hepatitis A, B, C, and E were all negative. Dengue and Malaria workups were also found to be negative. Ultrasound Pelvis revealed endometrial thickness measuring 8.9mm in the fundal part. However, in the lower uterine segment, it measured 11.5mm indicating a possibility of the retained product of conception (RPOC). An electrocardiogram (ECG) was also done which revealed sinus tachycardia. Echocardiography depicted an average-sized left ventricle with mild-to-moderately reduced left ventricular systolic function, a left ventricle ejection fraction (LVEF) of 40%, and grade I left ventricular diastolic dysfunction. Chest X-ray showed ground glass opacification seen in the mid and lower zones bilaterally. Additionally, the costophrenic angle appeared blunted by dense shadow due to mild pleural effusion. An impression of purpureal sepsis leading to DIC/TTP/HELLP syndrome was made. She was hydrated with normal saline and started on IV antibiotics, Meropenem 500mg BD and Vancomycin 1 gm 48 hourly. HCO3 was replaced as 200 mEq in 800 5% Dextrose water. Furthermore, she was given an injection of Transamine 1 gm TDS and an injection of Solumedrol 50mg 6 hourly. The patient was scheduled for manual vacuum aspiration (MVA) wherein the mild amount of RPOC and infected blood clots were removed, and she was subsequently transfused one packed cell volume (PCV), six fresh frozen plasma (FFP), and two platelet bags. A peripheral blood smear obtained showed schistocytes at 4.5%. Her urine output was 60–70ml per hour.

CBC was repeated as shown in Table 1. She was transfused three more PCVs, four platelets, and six FFPs accordingly. Meanwhile, her BUN and Creatinine continued to deteriorate, as well as the LFTs. Even though her urine output was adequate, BUN and Creatinine were on a raising pattern, and she was increasingly becoming agitated and delirious. She also developed sub-conjunctival hemorrhages. A nephrology opinion was obtained, and it was advised to consider the patient for hemodialysis. Three-hour hemodialysis was done with a BFR (blood flow rate) of 250 ml without Heparin in ICU by dialysis technicians as advised by the nephrologist.

Moreover, the urine culture sent came out positive for Enterococcus which was sensitive to Fosfomycin and Tigecycline. The former was added as 3 gm per oral for three days. Meropenem was stopped and Tigecycline 50 mg IV OD was started. The patient also developed peripheral pitting edema and mild abdominal distension with audible gut sounds. IV hydration was revised, and she continued to have three more sessions of hemodialysis. This was followed by four sessions of Plasmapheresis, performed by plasmapheresis technicians, each session 24 hours apart. The patient's platelet count as shown in Table 3, as well as BUN and creatinine, improved over 3–4 days with an improvement in delirium. She was subsequently stepped down to HDU and later discharged. She is being followed in OPD with satisfactory recovery and counseled for relapse in future pregnancies.

**Table 3:** Patients Platelet Count (PP = plasmapheresis).

Platelets	Day 1	Day 2	Day 3	Day 4	Day 5	Day 6	Day 7	Day 8
**Count**	19	13	47	12	27	15	61	158
**Intervention**	-	Post-transfusion	Post-transfusion	-	PP-1	PP-2	PP-3	PP-4

## 3 Discussion

Thrombotic microangiopathies can be life-threatening during pregnancy and the postpartum period. They encompass a group of disorders that have overlapping signs and symptoms, resulting in diagnostic challenges and timely intervention. Among these, rare TMA disorders viz TTP and atypical HUS can be mistaken for preeclampsia and HELLP syndrome delaying the recovery process of the patient [1].

In this study, we report a case of a 27-year-old female developing TTP and AKI following puerperal sepsis. The global incidence of puerperal sepsis ranges from 2.7 to 5.7%, leading to almost 2% mortality [6]. Our patient presented with vaginal bleeding along with the presence of vomiting, drowsiness, tachycardia, tachypnea, leukocytosis, thrombocytopenia, and systemic manifestations like chest pain, shortness of breath, and ventricular diastolic dysfunction, that may be attributed to the septic state [7]. Also, the ultrasonography results revealed retained products of conception (RPOC). Based on the clinical presentation and laboratory investigation findings, a preliminary diagnosis of puerperal sepsis was made. To eliminate the RPOCs and infected blood clots from the uterus, Mechanical Vacuum Aspiration (MVA) was performed. The WHO advises the use of MVA for the evacuation of RPOC and is a comparatively safe and less economically burdening procedure, especially for low-to-middle income countries with limited resources like Pakistan [8]. To compensate for the lost blood cells, platelets, electrolytes, and volume, the management was supplemented with infusions of PCV, FFP, platelets, HCO3- and normal saline rehydration therapy. For the treatment of sepsis, both broad and narrow-spectrum antibiotics i.e., Meropenem and Vancomycin, targeting both gram-negative and positive organisms were employed. Solu-Medrol, a synthetic glucocorticoid, was added alongside as an immunosuppressive and anti-inflammatory agent to overcome the septic state, while Transamine, (tranexamic acid) was administered to reduce bleeding. While transfusions helped to transiently increase the cell count, Hemoglobin, and platelet count, their levels fluctuated, as depicted in Table 1, Table 3

A urine sample was sent for culture and sensitivity and revealed an enterococcal infection sensitive to Fosfomycin and Tigecycline. Based on this, the treatment regimen was updated with Fosfomycin and Tigecycline and Meropenem was stopped. Although Escherichia. Coli, Klebsiella, and Staphylococcus Aureus remain the cornerstone of puerperal infections, Enterococcus is not commonly observed accounting for less than 2% of the cases [9]. The WHO recommends midstream urine and blood samples for the investigation of puerperal sepsis [10]. However, a blood sample was not implicated in this study, hence potentially limiting an effective culture result.

The association between sepsis and micro-thrombosis is well-appreciated. As a consequence of multiple molecular interactions and complement activation and subsequent Membrane Attack Complex (MAC) formation, it results in endothelial cell damage stimulating the ultra-large Von Willebrand factor (ULVWF) and tissue factor (TF) pathways, hence causing micro-thrombogenesis and TTP [11]. In the above-discussed case, the findings of thrombocytopenia, and schistocytes due to microangiopathic hemolytic anemia, anemia, renal deficit, neurologic manifestations, and subconjunctival pinpoint hemorrhages supported the diagnosis of TTP. Despite adequate urinary output, the BUN and creatinine levels escalated indicating an acute kidney injury compromising the activity of the organs. The patient also became agitated and delirious, potentially stemming from elevated urea levels. Thus, she was subsequently placed on hemodialysis to improve renal function.

Plasma exchange therapy remains the mainstay treatment for TTP [12], with our patient undergoing 4 sessions, each 24 hours apart with subsequent clinical improvement and complete recovery. Timely modifications in the treatment regimen enabled a complete recovery, and the patient was soon discharged.

Several cases of postpartum-associated TTP have been reported in the literature, with varying outcomes [4]. Shamseddine et al. reported four cases of pregnancy-related TTP in which appropriate treatment ensured complete recovery in two cases, while the remaining two culminated in complications leading to maternal death and intrauterine growth retardation resulting in intrauterine fetal death. While puerperal infections account for significant morbidity and mortality, appropriate measures can help reduce their incidence substantially. It accounts for 10–12% of maternal mortality in developing countries, relative to the developed nations where sufficient measures enabled a reduction to 2% [5]. Hence, it is imperative to report cases of pregnancy-associated TTP and multiple organ involvement with a particular emphasis on treatment regimens and outcomes to raise awareness regarding the successful management of such complications among clinicians and consequently, alleviate maternal and fetal mortality.

Apart from TTP, several other pregnancy-related conditions may involve thrombotic microangiopathies (TMA) with pre-eclampsia, HELLP (Hemolysis, elevated liver enzymes, low platelets) syndrome, and hemolytic uremic syndrome being the major culprits. To prevent any negative pregnancy outcomes, it is imperative to timely diagnose and treat thrombotic disorders [1]. Recognition of at-risk patients may further help health care professionals prepare for any complications particularly in females with a previous history of pregnancy-related TMAs, diagnosed cases of immune thrombotic thrombocytopenia (ITP), and hereditary TTP. It is also imperative to follow up on these patients and keep them on surveillance for any relapse of microangiopathy in future pregnancies [13].

## 4 Conclusion

TTP is an uncommon thrombotic microangiopathy seen in the intrapartum and postpartum periods. It may be preceded by puerperal sepsis but needs timely intervention to prevent mortality and complete recovery. Extra vigilance may be required to monitor patients for acute kidney injury, psychosis, and thrombocytopenia. Surveillance is necessary in future pregnancies to avoid its relapse.

## Ethical Approval

Ethical approval from the relevant hospital department was obtained.

## Sources of Funding

The authors declare no source of funding.

## Author contribution

Bushra Majid: Study conception, manuscript drafting, critical review, Syed Hassan Ahmed: Literature search, manuscript drafting, Hanifa Majid: Literature search, manuscript drafting.

## Declaration of competing interest

The authors declare no competing interest.
